# Parental physical disease severity and severe documented physical child abuse: a prospective cohort study

**DOI:** 10.1007/s00431-023-05291-8

**Published:** 2023-10-27

**Authors:** Troels Græsholt-Knudsen, Charlotte Ulrikka Rask, Steven Lucas, Carsten Obel, Bodil Hammer Bech

**Affiliations:** 1 Research Unit for Mental Public Health, Department of Public Health, Aarhus University, Bartholins Allé 2, 8000 Aarhus C, Aarhus, Denmark; 2 Department of Forensic Medicine, Palle Juul-Jensens Boulevard 99, Aarhus University, 8200 Aarhus N, Aaurhus, Denmark; 3 Department of Child and Adolescent Psychiatry, Aarhus University Hospital Psychiatry, Palle Juul-Jensens Boulevard 175, 8200 Aarhus N, Aarhus, Denmark; 4 Department of Clinical Medicine, Aarhus University, Olof Palmes Allé 43, 8200 Aarhus N, Aarhus, Denmark; 5 Department of Women’s and Children’s Health, Uppsala University, Akademiska sjukhuset, 751 85 Uppsala, Sweden; 6 Research Unit for Epidemiology, Department of Public Health, Aarhus University, Bartholins Allé 2, 8000 Aarhus C, Aarhus, Denmark

**Keywords:** Child, Maltreatment, Physical child abuse, Parental health, Risk factor, Causal

## Abstract

Successful prevention of physical child abuse is dependent on improvements in risk assessment. The risk of abuse is assumed to increase when family stressors overcome resources. Severe physical disease can increase stress, and parental physical disease has been studied as a risk factor for physical child abuse, but with heterogeneous definitions. This study evaluated the relation between parental physical disease severity and severe documented physical child abuse. Models were based on data on children aged 0–17 years in Denmark between 1997 and 2018, and their parents. Severe documented physical child abuse was modeled as violence against a child registered by either health authorities in treatment or mortality registries, or police authorities in cases confirmed by the courts. Parental physical disease severity was modeled as the sum of Charlson Comorbidity Index scores for the child’s parents. The causal connection was examined in two model types: a survival model comparing exposed with non-exposed children, adjusted for covariates at baseline, and a G-model, taking time-varying covariates, including income and parental psychiatric disease into account. Neither model showed an association between parental physical disease severity and severe documented physical child abuse, with RR 0.99 and 95% CI (0.93–1.05) for the survival model and RR 1.08 for the G-model (CI not calculated).

*  Conclusion*: In the model studied, parental physical disease severity was not a risk factor for severe documented physical child abuse. Individual categories of physical disease remain to be examined.

*  Trial registration*: The study was pre-registered on Open Science Framework, https://osf.io/fh2sr.

**Table:** 

**What is Known:** *• Parental physical disease severity has been studied previously as a risk indicator of physical child abuse, but based on heterogeneous definitions.* *• Previous studies have not studied parental physical disease severity preceding physical child abuse.*	
**What is New:** *• Parental severe physical disease was not prospectively associated with severe documented physical child abuse in a survival model, a G-model and a number of sensitivity analyses, respectively.* *• Results should be replicated in samples from populations without universal health care, and using different categories of disease.*

**Supplementary Information:**

The online version contains supplementary material available at 10.1007/s00431-023-05291-8.

## Introduction

Physical child abuse is detrimental to child well-being and has adverse consequences throughout life [1–5]. Successful prevention necessitates proper risk assessment and early intervention, and a number of tools exist for early clinical detection [6–8]. Knowledge on causes and risk factors for physical child abuse is used to develop such interventions. Despite the often cited causal assumption that physical child abuse occurs when family resources are overwhelmed by stressors [9], to the best of our knowledge, causal inference techniques and theory [10] have only been used sparsely in the field of physical child abuse [11], although this has been encouraged [12]. As improvements in preventive efforts are called for [13], research is warranted to define which risk factors are most pertinent, including parental factors that are likely to induce stress in the whole family. Physical disease in adulthood is known to cause perceived individual stress [14]. However, whereas psychiatric disease among parents has previously been shown to increase the risk of lethal physical child abuse [15, 16], parental physical disease has scarcely been studied as a risk factor. Within the highly diverse construct of physical disease, only a few definitions have been explored. Chang et al. found an association to child maltreatment, but they did not ensure that parental physical disease preceded child maltreatment temporally [17]. Household AIDS, but not other household chronic illness, predicted a high intensity of abuse [18]. “Poor maternal health” was associated with physical child abuse but was not further defined, and the temporal separation of parental health and child abuse was not clear [19]. Thus, although there are indications in the literature that parental physical health is a predictor of subsequent physical abuse, the temporality, robustness, and dose–response relationship between parental physical disease and physical child abuse remains unclear.

This study aims to explore the causal link between parental physical disease severity and severe documented physical child abuse. We hypothesize that parental physical disease severity, measured using a joint Charlson score [20] and preceding reported first incidents of abuse, will indicate the risk of severe physical child abuse documented in official registries.

## Methods

### Data and design

All public authorities in Denmark use the same unique personal identifier across registries [21]. This was used to create a prospective data set by linking administrative data on all children living in Denmark from the 1st of January 1997 until the 31st of December 2018 and their legal parents, updating the dataset using monthly intervals and using an existing data framework [22]. The year 1997 was chosen because some data sources used were initiated in this year, and 2018 was the latest available year, maximizing available data for a rare outcome. Children lacking both parents were censored from the first time point of this event. As the personal identifier is a prerequisite for citizenship and many public services, the coverage in the population is assumed to be practically complete. This study has been pre-registered, available along with changes since pre-registration and project code [23]. Access to data was granted by Statistics Denmark, and this study was exempt from ethics approval according to Danish law, as it only used administrative data.

### Exposure—parental physical disease severity

Parental physical disease severity was modeled using a sum of both parents’ Charlson Comorbidity Index [20] as a proxy to joint parental stress from physical disease severity. Data for the index were drawn from the Danish National Patient Register. The Charlson Comorbidity Index is known as a reliable index of long-term mortality and is widely used in clinical settings [24]. ICD 8 and 10 diagnoses that increased the Charlson Comorbidity Index [25] for an individual parent were assigned points according to the original definition [20], and all diagnoses available 5 years back [25] from any given month in the dataset and until physical child abuse or censoring were included in the calculation. A full description is provided in Table 1.

**Table 1:** Scoring of Charlson Comorbidity Index

Category	ICD-10 codes	ICD-8 codes	Scoring weights
Myocardial infarction	I21, I22	410	1
Heart failure	I099, I110, I130, I132, I255, I425, I426, I427, I429, I428A, P290, I43, DI50, DE105, DE115, DE125, DE135, DE145	42709, 42710, 42711, 42719, 42899, 78249	1
Peripheral vascular disease	I70, I71, I72, I731, I738, I739, I77, I790, I792, K551, K558, K559, Z958, Z959	440, 441, 442, 443, 444, 445	1
Cerebrovascular disease	I60, I61, I62, I63, I64, I65, I66, I67, I68, I69, G45, G46, H340	430, 431, 432, 433, 434, 435, 436, 437, 438	1
Dementia	F00, F01, F02, F03, G30, F051, G311	290	1
Chronic pulmonary disease	J40, J41, J42, J43, J44, J45, J46, J47, J60, J61, J62, J63, J64, J65, J66, J67, J684, I278, I279, J84, J701, J703, J920, J953, J961, J982, J983	515, 516, 517, 518, 490, 491, 492, 493	1
Rheumatic disease	M05, M06, M08, M09, M30, M31, M32, M33, M34, M35, M36, D86	712, 716, 734, 446, 13599,	1
Peptic ulcer disease	K25, K26, K27, K28, K221	53091, 53098, 531, 532, 533, 534	1
Mild liver disease	B18, K700, K701, K702, K709, K703, K713, K714, K715, K717, K73, K74, K760, K762, K763, K764, K769, Z944	571, 57301, 57304	1
Severe liver disease	B150, B160, B162, B190, I850, I859, I864, I982, K704, K711, K721, K729, K765, K766, K767	07000, 07002, 07004, 07006, 07008, 57300, 45601, 45602, 45603, 45604, 45605, 45606, 45607, 45608, 45609	3
Diabetes without complications	E100, E101, E108, E109, E110, E111, E119, E120, E121, E129, E130, E131, E139, E140, E141, E149	24900, 24906, 24907, 24909, 25000, 25006, 25007, 25009	1
Diabetes with complications	E102, E103, E104, E105, E106, E107, E112, E113, E114, E115, E116, E117, E118, E122, E123, E124, E125, E126, E127, E128, E132, E133, E134, E135, E136, E137, E138, E142, E143, E144, E145, E146, E147, E148	24901, 24902, 24903, 24904, 24905, 24908, 25001, 25002, 25003, 25004, 25005, 25008	2
Hemiplegia/paraplegia	G81, G82, G830, G831, G832, G833, G834, G041, G114, G801, G802, G839	344	2
Renal disease	N032, N033, N034, N035, N036, N037, N052, N053, N054, N055, N056, N057, Z490, Z491, Z492, N18, N19, I120, I131, I132, N250, Z940, Z992, N26	403, 404, 580, 581, 582, 583, 584, 59009, 59319, 75310, 75311, 75312, 75313, 75314, 75315, 75316, 75317, 75318, 75319, 792	2
Any malignancy	C0, C1, C2, C3, C40, C41, C42, C43, C44, C45, C46, C47, C48, C49, C5, C6, C70, C71, C72, C73, C74, C75, C76, C86, C97	140, 141, 142, 143, 144, 145, 146, 147, 148, 149, 150, 151, 152, 153, 154, 155, 156, 157, 158, 159, 160, 161, 162, 163, 164, 165, 166, 167, 168, 169, 170, 171, 172, 174, 175, 176, 177, 178, 179, 180, 181, 182, 183, 184, 185, 186, 187, 188, 189, 190, 191, 192, 193, 194, 27559	2
Metastatic solid tumor	C77, C78, C79, C80	195, 196, 197, 198, 199	6
AIDS/HIV	B20, B21, B22, B23, B24	07983	6
Leukemia	C91, C92, C93, C94, C95	204, 205, 206, 207	2
Lymphoma	C81, C82, C83, C84, C85, C88, C90, C96	200, 201, 202, 203, 27559	2

The Charlson Comorbidity Index was originally devised to predict mortality in longitudinal studies and has been used extensively in the literature, with the original article describing the index currently cited 42,206 times [20]. Its predictive validity for long-term mortality has been tested in diverse populations [24]. We used the original classification with 19 categories, and the original weights for scoring as described by the team behind the Heaven package [25]. The index was calculated individually for the parent(s) and summed to give a joint index. As two parents could get higher scores than one, a sensitivity analysis did analyses separately for single parents and non-single parents; see sensitivity analyses. Mild and severe liver disease, diabetes with and without complications, and any malignancy and metastatic solid tumor are mutually exclusive

### Outcome variable

Severe documented physical child abuse was identified through three sources: the Danish National Patient Register [26], the Cause of Death Register [27], and the Danish Criminal Register. All models in this study focused on first-time events. All children with events before the inclusion date were excluded. Severe documented physical child abuse was operationalized as abuse that resulted in contact with a hospital, including the emergency department or any other department coding as described below. Also, death of the child resulting from non-accidental injuries or a court sentence of the perpetrator describing violence according to police codes was included in the outcome. Coding for documented physical abuse included all codes recommended by the Danish Paediatric Society [28], and all codes that specified physical violence (not accidents or uncertain intent) towards any individual less than 18 years of age. For details on codes and diagnoses used, see pre-registration and associated resources [23].

### Covariates

**Fig. 1:**
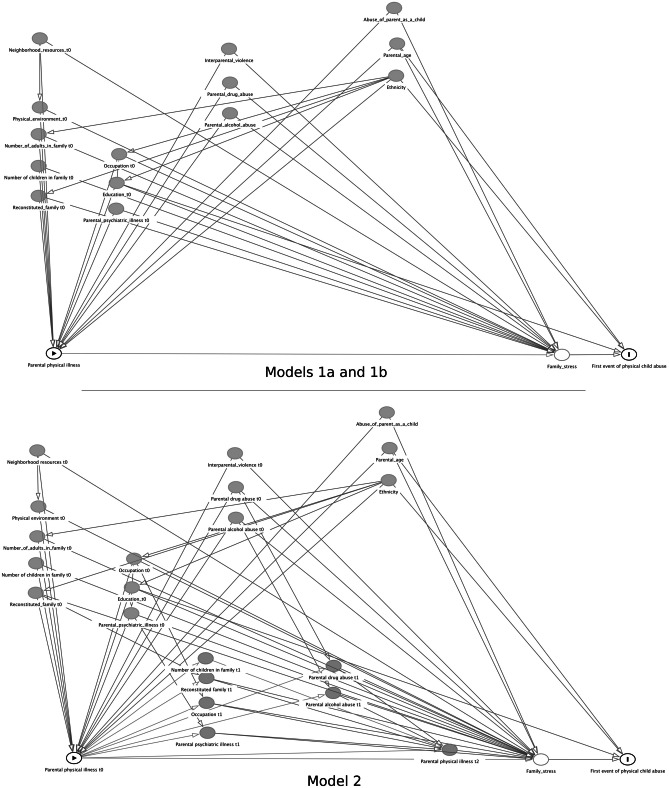
These directed acyclic graphs depict the causal pathways assumed in our models. Except for physical environment, all other variables are adjusted for; income is used as a proxy for occupation. For models 1a and 1b, the variables are measured at baseline and constant during risk time, reflecting an assumption that the variables after baseline may be mediators. For model 2, the exposure is allowed to affect covariates, which in turn affect the exposure. For simplicity, only T0, 1, and 2 are shown here

Variables were chosen based on two Directed Acyclic Graphs [10], printed here as Fig. 1. They were drawn with exposure and outcome in mind, and based on prior literature, see Table 2. The link between ethnicity and physical child abuse was based on prior literature, showing differing risks for physical child abuse after adjusting for covariates [29–32]. Except where otherwise noted, variables were programmed as defined in the pre-registration. Table 2 provides references to further descriptions of the registries used.

**Table 2:** List of variables, their study definitions, sources, and levels

**Variable**	**Registry source**	**Study definition**
Child age	Fertility Database [33], Population Register, Medical Birth Register [34]	Child age at inclusion, as recorded in population registers
Calendar year group		Calendar year group at inclusion
Mean parental age	Fertility Database, Population Register, Medical Birth Register	Parental or guardian age at inclusion; in case of more than one parent or guardian, the mean of ages was computed. In model 2, as legal guardians could change during study participation, this is treated as a time-varying covariate
Neighborhood resources	Income Register [35], Parish Register, Population Register, Address Register, Addresses in Denmark Register (except for Parish Register, the remaining registers are all connected to the population register)	Neighborhood homogeneity as measured from family total income, inspired by Cheung et al. [36]. The variable was in thousands, the difference between the highest and the lowest quartile of average family income through the last three years, among families with children in the parish, or clustering of parishes according to another study [37], and subtracting the yearly rate for relative poverty [38, 39]
Number of children in family^a^	Population Register, Central Personal Register Status	The number of other children with the same parent(s) as the child, inspired by multiple studies [32, 40, 41]
Maltreatment of parent as a child^b^	Children and Young Adults (preventive actions) Register, Danish National Patient Register and related^c^, Victims of Criminal Offences Register [42]	Welfare services interventions, child health records and criminal records joined in a variable indicating presence of either maltreatment or neglect, inclusion inspired by Widom et al. [43]
Immigration background (ethnicity)	Fertility Database, Population Register [21], Residence Permit Register [44]	Country of birth or citizenship for parents and children, immigrant was defined as at least one parent originating from a foreign country, inclusion was inspired by multiple studies [29–32]
Status as refugee	Residence Permit Register	The familial basis of residence in Denmark, as available in the registries; any family granted permanent or temporary residency as refugees, based on the right to asylum or similar, or residency based on humanitarian grounds or risk assessment, were classified as refugees. This variable was included based on expert opinion (H.N. Christensen, personal communications, 2019)
Reconstituted family	Fertility Database, Population Register	Whether child was living with biological parents only, was adopted, or living with a stepparent—this variable, along with the connection between child and parents, was updated each year instead of each month, inclusion was inspired by Schnitzer et al. [45]
Education^d^	Education Register [46]	Highest level of finished education among the parents, the ISCED classification was used to make a classification of the highest educated adult in the household, inclusion inspired by multiple studies [32, 40]
Income	Income Register	Mean family income during last three years as a continuous variable measured in hundreds, subtracting the yearly rate for relative poverty [38] so that a negative income meant an income less than the current poverty level in that year, inclusion inspired by Paxson et al. [47]
Parental psychiatric disease^d^	Danish National Patient Register and related^c^	Diagnosis of psychiatric disorders or dispensation of medication for psychiatric disorders, drawn from the patient register and the register of drug prescriptions. This was a cluster of variables, as described by Prior [14], and modified, adding personality disorder as described in the algorithm for exposure. Psychological distress was not used because of a broad definition. The categories for alcohol problems and other substance abuse was described separately. Inclusion was inspired by multiple studies [15, 16]
Inter-parental violence	Danish National Patient Register and related,^c^ Victims of Criminal Offences Register	Observations of violence by the police in conjunction with health data on injuries. This was a dichotomous variable indicating whether or not inter-parental violence has ever taken place and could go from negative to positive, but not back to negative, as inter-parental violence is known to have effects on risk lasting several years [48]. For codes used, see code [23]. Inclusion inspired by Jobe-Shields et al. [49]
Parental drug abuse^e^	Danish National Patient Register and related^c^	Any drug-related diagnosis and any crime related to drug possession or intake—was a dichotomous variable indicating abuse or no abuse. The literature does not carry precise instructions about whether former substance abuse carries increased risk at present [15, 50–52]. We chose a partly arbitrary limit of 2 years [14], to allow for presumed differing risks among current and former substance abusers, and the fact that ongoing substance abuse may occur with no indications in the registries. Classification was inspired by Ahacic et al. [53], and inclusion by Wasserman et al. [52]
Parental alcohol abuse^e^	Danish National Patient Register and related^c^	Any alcohol-related diagnosis and any crime related to alcohol intake—was a dichotomous variable indicating abuse or no abuse and going two years back, same arguments as for drug abuse, inclusion inspired by Gessner et al. [32]

The studies cited provided findings to justify the inclusion of the variables

^a^This variable was based on number of children with the same parents (including single parents) instead of the planned definition due to unexplained inaccuracies in the dataset. When using the original sources, in some instances, data indicated cohabitation of several hundred adults and children

^b^Registrations of maltreatment in ICD 8 is less detailed than ICD 10 [23]. This variable was collapsed to avoid small cells in the analysis

^c^Danish National Patient Register (in- and outpatient contacts) [26], causes of death register [27]

^d^This variable was collapsed to avoid small cells in the analysis

^e^These were collapsed into parental substance abuse to avoid small cells in the analysis

### Analysis

Model 1 assumed that the first change in parental physical disease severity could have downstream consequences on health and covariates. This was modeled as a survival model comparing exposed with unexposed children and analyzed by regression using pseudoobservations to estimate risk ratios [54]. Exposed children had joint parental Charlson score of two or more. Single parents were included with their own Charlson score. The index date was when the joint parental Charlson score reached two or more, or the child’s birth date, whichever came last. Exposed children were matched with 5 children with joint parental Charlson score less than two, matching on index date (matches drawn within 3 months), reconstituted family (living with biological parent(s), living with one or more unrelated adults, adopted or in foster care), child ages (within 1 year), number of children (according to categories), and mean parental age (difference less than or equal to 5 years). Pseudoobservations assumed that censoring in the dataset was marginally independent of the covariates. This was checked by fitting a Cox model of the covariates with censoring as the dependent variable. Calendar year was not marginally independent of censoring, and, adjusting for this, pseudoobservations were generated after stratifying on calendar year group. Model 1a presents the model as specified a priori. The influence of collinearity and small cells as a result of many covariates was controlled by removing candidates with collinearity and skewed distributions, shown as model 1b. The linearity of continuous variables was checked by introducing their quadratic terms in the model. Only cases with information on all variables were analyzed. For distribution across covariates, see Online Resource 1. The number of missing values across variables can be seen in Table 3. The same child could be included in the analysis as both exposed and non-exposed; to adjust for this, Eicker-Huber-White estimations were used for standard errors [55]. As siblings could be included, clustering was adjusted for at the family level, where each unique combination of parents, including single parents, was counted as a cluster. Children were followed from index date to outcome, emigration, death, age 18 years or the first of January 2018, whichever came first.

**Table 3:** Baseline characteristics of the source population according to severity of parental physical disease

Characteristics	Joint Charlson score < 2, *n*: 2,659,586	Joint Charlson score ≥ 2, *n*: 46,184	Total population *n*: 2,705,770
Child age at cohort entry, y, median (quartile 1; quartile 3)^a^	0.0 (0.0; 7.0)	0.7 (0.0; 11.3)	0.0 (0.0; 7.1)
Calendar year group, *n* (%)			
1997–2002	1,577,955 (59%)	27,619 (60%)	1,605,574 (59%)
2003–2009	501,203 (19%)	7210 (16%)	508,413 (19%)
2010–2018	580,428 (22%)	11,355 (25%)	591,783 (22%)
Mean parental age, y, mean (SD)	34.33 (6.61)	37.6 (7.41)	34.38 (6.64)
Missing entries	17,203 (0.7%)	57 (0.1%)	17,260 (0.6%)
Neighborhood resources, thousand euros, mean (SD)	106.72 (37.73)	109.01 (41.33)	106.76 (37.8)
Missing entries	546,043 (21%)	6208 (13%)	552,251 (20%)
Number of children in family, *n* (%)			
One child	1,226,120 (46.1%)	20,999 (45%)	1,247,119 (46%)
Two children	986,407 (37%)	17,635 (38%)	1,004,042 (37%)
Three to five children	435,873 (16%)	7269 (16%)	443,142 (16%)
Six or more children	11,186 (0.4%)	281 (0.6%)	11,467 (0.4%)
Parental maltreatment in childhood, *n* (%)			
No maltreatment or neglect	2,492,912 (94%)	43,116 (93%)	2,536,028 (94%)
Maltreatment or neglect, one or both parents	166,674 (6.3%)	3068 (6.6%)	169,742 (6.3%)
Immigration background (ethnicity), *n* (%)			
No foreign parents	1,760,272 (66%)	34,477 (75%)	1,794,749 (66%)
One or more foreign parents	580,749 (22%)	8820 (19%)	589,569 (22%)
Missing entries	318,565 (12%)	2887 (6.3%)	321,452 (12%)
Status as refugee, *n* (%)			
Not in need of protection	2,579,134 (97%)	45,293 (98%)	2,624,427 (97%)
In need of protection	80,452 (3.0%)	891 (1.9%)	81,343 (3.0%)
Reconstituted family, *n* (%)			
Living with biological parent(s)	1,931,986 (73%)	36,531 (79%)	1,968,517 (73%)
Living with one or more unrelated adults	72,173 (2.7%)	1747 (3.8%)	73,920 (2.7%)
Adopted or in foster care	39,249 (1.5%)	866 (1.9%)	40,115 (1.5%)
Missing entries	616,178 (23%)	7040 (15%)	623,218 (23%)
Family highest education, *n* (%)			
Primary or secondary education	1,458,294 (55%)	25,635 (56%)	1,483,929 (55%)
Tertiary education or higher	913,758 (34%)	17,152 (37%)	930,910 (34%)
Missing entries	287,534 (11%)	3,397 (7.4%)	290,931 (11%)
Family income, thousand euros, mean (SD)	123.02 (245.55)	128.8 (135.56)	123.13 (243.99)
Missing entries	360,292 (14%)	3403 (7.4%)	363,695 (13%)
Parental psychiatric disease, *n* (%)			
No psychiatric disease	2,566,405 (97%)	43,350 (94%)	2,609,755 (96%)
Any psychiatric disease except substance abuse	93,181 (3.5%)	2834 (6%)	96,015 (3.6%)
Inter-parental violence, *n* (%)			
No interparental violence	2,658,887 (99%)	46,165 (99%)	2,705,052 (99%)
Interparental violence	699 (0.0%)	19 (0.0%)	718 (0.0%)
Parental substance abuse, *n* (%)			
No parental substance abuse	2,638,446 (99%)	44,858 (97%)	2,683,304 (99%)
Any parental substance abuse	21,140 (0.8%)	1326 (2.9%)	22,466 (0.8%)

Baseline is the first observation for each individual participating. This is also true for the joint Charlson score, the children described here with a parental score ≥ 2 are those entering into the sample of 2,705,770 children with this status. Online Resource 1 describes the combination of these children and those who become exposed during the study

^a^Rounded to first decimal

Model 2, in Fig. 1, assumed that family physical disease severity could influence and be influenced by covariates during the child’s contributed risk time. This was modeled as a G-model [10]. For this model, imputation was performed [56] and analyses were carried out on a reduced dataset with half-year intervals and a random draw of 50,000 children; see Online Resource 2. Imputation convergence and distribution of resulting variables were controlled with plots of iteration means and standard deviations, density, and box plots of distributions [57], and for the categorical and binary variables, the distribution of imputed values was compared to the observed, inspired by Harrell; results not shown [58].

## Results

The characteristics of the full population, from which the samples for model 1 and model 2 were drawn, are shown in Table 3.

### Model 1a and 1b: survival model

Data from 1,160,840 children were used, 43% of the original cohort, and after removing those that could not be matched with five unexposed controls, 1,160,529 children remained. The average follow-up time for these children was 12.22 years, and the total follow-up time was 14,184,982 years. In this dataset 28,190 children were registered as having been exposed to documented physical abuse for the first time between 1997 and 2018, including 29 cases of lethal abuse. Among the 1,160,529 children, deaths with no known relation to physical abuse during the same period totaled 1097. Model 1a showed a relative risk for children with parental joint Charlson score of two or more of 0.99 (0.93–1.05) and model 1b of 1.02 (0.96–1.08). As the Directed Acyclic Graph was designed with parental physical disease severity in mind, all other estimates in Table 4 cannot be interpreted causally but are reported for comparison with the existing literature. A parent with tertiary level education or higher predicted less abuse with a relative risk of 0.53 (0.50–0.57) (model 1a) or 0.53 (0.49–0.56) (model 1b) compared to primary education, respectively, and parental history of childhood maltreatment or neglect showed a relative risk of 2.25 (2.10–2.40) (model 1a) or 2.30 (2.16–2.46) (model 1b). See Table 4 for further information.

**Table 4:** Results from survival models 1a and 1b

Variable	Relative risk, model 1a^a^	*P*-value	Relative risk, model 1b^a^	*P*-value
(Intercept)	0.11 (0.10–0.12)	< 0.001	0.11 (0.10–0.12)	< 0.001
Joint Charlson Score				
< 2				
≥ 2	0.99 (0.93–1.05)	0.638	1.02 (0.96–1.08)	0.619
Family income, hundred euros	1.00 (1.00–1.00)	< 0.001	1.00 (1.00–1.00)	< 0.001
Neighborhood resources, hundred euros	1.00 (1.00–1.00)	0.322	1.00 (1.00–1.00)	0.886
Immigration background (ethnicity)				
No foreign parents				
One or more foreign parents	1.30 (1.20–1.41)	< 0.001	-	
Status as refugee				
Not in need of protection				
In need of protection	1.13 (0.85–1.50)	0.390	-	
Calendar time group				
1997–2002				
2003–2009	0.66 (0.62–0.70)	< 0.001	0.66 (0.62–0.70)	< 0.001
2010–2018	0.25 (0.23–0.28)	< 0.001	0.25 (0.23–0.27)	< 0.001
Family highest education				
Primary or secondary education				
Tertiary education or higher	0.53 (0.50–0.57)	< 0.001	0.53 (0.49–0.56)	< 0.001
Parental psychiatric disease				
No psychiatric disease				
Any psychiatric disease	1.31 (1.20–1.42)	< 0.001	1.42 (1.32–1.53)	< 0.001
Interparental violence				
No interparental violence				
Interparental violence	2.07 (1.61–2.65)	< 0.001	-	
Parental substance abuse				
No parental substance abuse				
Parental substance abuse	1.40 (1.25–1.56)	< 0.001	-	
Parental maltreatment in childhood				
No maltreatment or neglect				
Maltreatment or neglect, one or both parents	2.25 (2.10–2.40)	< 0.001	2.30 (2.16–2.46)	< 0.001

The Directed Acyclic Graph underlying this study was developed for the joint Charlson score and thus is the only variable that should be interpreted causally. Other variables were included to ensure adjustment for confounding, and their coefficients are presented for comparison purposes with other studies

^a^Model 1a was adjusted for all the variables shown and was specified a priori. Model 1b was specified after analyzing collinearity and small cells in the categorical variables; the variables marked with a dash (-) are left out

### Model 2: G-model

Plots of imputed data compared to observed data showed plausible results for all five datasets. The G-model on a random extract of 50,000 children from the main dataset showed that parental joint Charlson score of two or more had a relative risk of 1.08 for documented child abuse compared to unexposed children, adjusting for the same variables as included in model 1 with the exception of interparental violence, parental substance abuse, and status as refugee, as the model could not converge with the full set of variables.

### Sensitivity analyses

Because of computational intensity, all model control and sensitivity analyses were carried out on variations of model 1, except for control of pseudoobservations which was done in a Cox model. Model results are not shown. The introduction of quadratic terms was significantly associated with the outcome but did not influence the main result. A number of separate models excluding parents with a registered death before the end of the study period, excluding child deaths, stratifying on less than and older than 7 years, changing the joint Charlson score cutoff to 4 and 8 (two different models), excluding immigrants and emigrants, and stratifying on children living with single parents and children living in other family arrangements showed essentially unchanged results (data not shown). A model exchanging parental physical disease with psychiatric disease, leaving out parental physical disease, showed a RR of 1.41 (1.30–1.52). Part of the outcome was drawn from the Victims of Criminal Offences registry, only available from after 2000, and restricting the data to this time frame, results were essentially unchanged (data not shown).

## Discussion

This study aimed to explore the link between parental physical disease severity and severe documented physical child abuse. The results from all models correspond closely and do not indicate a causal connection. To the best of our knowledge, there are no similar longitudinal results in the literature for comparison. However, given the methodological challenges mentioned in the introduction with preceding studies, this result is not in opposition to earlier findings. The associations estimated for the covariates from model 1a do not allow causal inference, but the associations demonstrated were in line with previous literature, including the negative association with family highest education [32], and the positive association with parental maltreatment in childhood [43]. The sensitivity analysis exchanging the exposure with parental psychiatric disease also showed an association with parental psychiatric disease, in line with earlier studies [15, 16].

### Strengths and weaknesses

To the best of our knowledge, no study before this has tested a causal hypothesis on parental physical disease and severe documented physical child abuse. Models and all definitions of variables were based on prior literature, and their associations were illustrated in a pre-specified directed acyclic graph. Also, no prior study on parental physical disease has tested disease severity as a risk factor. This study uses data from health registries, and police cases confirmed by the courts. Data from social services were not available on a national level in sufficient detail to be included in this study. This is likely to limit cases to those severe enough to warrant either hospital treatment, sufficient evidence for a court trial, or both. Consequently, our results may not be generalizable to the large number of cases of physical child abuse that do not reach the attention of health care or law enforcement. Because of the computational intensity of the models, the number of children included was limited by available machine power, even on a server with 1 terabyte of working memory. For the same reason, as imputation in the full dataset was not feasible, only cases with information on all variables were used in model 1, thereby deviating from the pre-registration. The number of missings across variables was substantial. Assuming that low socioeconomic status predicts an increased risk of both missing variables [59], parental physical disease [60], and physical child abuse [29], bias could be introduced. However, this would be accounted for by adjusting for socioeconomic status. As we adjust for both level of education and income, the influence of this bias is expected to be small. Additionally, finding similar results across two models based on two different data excerpts, one of which used imputation for missing values, and finding essentially unchanged results, does not indicate major influence by missing values. This also indicates a minimal risk of major differences when using a larger dataset or computer. The population-level data on place of residence proved to be unreliable, to the point that the number of adults in each family could not be reliably determined, and this was a deviation from the pre-registration. However, the reconstituted family variable made a useful replacement and is assumed to explain at least part of the same variation. Who lives with the child is of more importance than how many adults are in the household. The data specified if a child lived with both biological parents, with a step-parent, or if the child was adopted or in foster care. Living with a single parent may have influenced the joint Charlson score. However, a sensitivity analysis stratifying the dataset on single parents and children living in other family constitutions showed essentially unchanged results. The G-model includes a large number of model specifications, thus increasing the risk of mis-specifications. Nonetheless, it is reassuring that the G-model and the regression based on pseudoobservations reach highly similar conclusions, despite very different assumptions. The median child age at index date in the source population (Table 3) is 0.0, 0.4 in the random draw from the source population (Online Resource 2), and 10.3 in data for model 1 (Online Resource 1). The age differences between samples question the comparability between model 1 and 2. It is reassuring that stratifying model 1 into children younger than and older than 7 years gives essentially unchanged results in both strata, and that the results in model 1a, 1b, and 2 are similar. Confidence intervals for the G-model were intended to be derived from bootstrapping by family unit to adjust for similarities among siblings. However, because of the reduced sample size of 50,000 and only 1.7% of families with joint Charlson scores above 2, the G-model did not converge reliably using the bootstrapped samples. Therefore, model 2 was presented without confidence intervals. For both models 1 and 2, the few families with a joint Charlson score above 2 meant a small group of exposed children, indicating that the Charlson score might not appropriately describe disease in the parental population.

The study has controlled for socioeconomic indicators and age and is comprehensive in the inclusiveness of all kinds of citizens in Denmark and can thus be generalized to similar populations with some degree of certainty. A note of caution should be made to societies without universal health care, as it seems likely that disease in such a society may affect the family stress level differently than in this cohort. The access to affordable health care has been shown to affect levels of maltreatment, although not physical abuse [61]. Another issue is that data from administrative sources largely underestimate the incidence of physical child abuse [62]. Thus, misclassification of outcomes as non-abuse could result in a weakening of any associations found in the present study. As there is a risk that the underlying populations differ, our results may not be generalizable to cases of physical child abuse unknown to authorities or, as mentioned above, known only to child protective services. We had access to data on the entire child population living permanently in Denmark up until 2018 in this study, and in models 1a and 1b, we used substantial samples of this population. We did, however, not use a population correction factor to correct our confidence intervals. This was because we regarded the population studied as a sample of a child population living with universal health care, which is a limited population but a population extending beyond Danish children. Using a population correction factor on models 1a and 1b would have narrowed their confidence intervals but likely not have changed their interpretation, as they are both close to 1 with point estimates 0.99 and 1.02. Model 2 was run on only a subsample of 50,000 children and would therefore not justify the use of a population correction factor.

### Interpretation

The results fail to reject the null hypothesis. This could indicate that although parents may be stressed by documented physical disease [14], this stress does not result in an increased risk of severe documented physical child abuse. It is, however, also possible that specific entities of disease are associated with documented physical abuse in line with the hypothesis, but that any signal from these are muted by a large heterogeneity in the diseases included in the Charlson index. Different categories of disease would have to be studied separately to uncover this. In addition, to better describe this population, a definition of physical disease not emphasizing severity but broader disease categories might show different results, as high Charlson scores are rare in this population, affecting only 1.7% of families. Furthermore, levels of health-related stress may differ in families living in countries without universal health care, and results may not replicate in such settings. If longitudinal results differ in such countries, our results might indicate that access to health care in families with parental physical disease is protective against physical child abuse.

## Conclusion

The present data showed no link between parental physical disease and an increased risk of severe documented physical child abuse. A possible explanation is that previous studies have not modeled disease severity, categories, and covariates in a similar cohort. Other explanations could be that any disease categories consistent with the hypothesis are muted by disease heterogeneity. Further research should explore individual disease categories and seek to confirm results in countries with non-universal health care, and in data sets including cases of physical abuse unknown to authorities, as the results might differ in these populations. Also, these results should be confirmed in data with self-report of physical child abuse.

### Supplementary Information

Below is the link to the electronic supplementary material.

Supplementary file1 (DOCX 8 KB)

Supplementary file2 (DOCX 10 KB)

## Data Availability

Data could not be made available due to legal requirements by Statistics Denmark.

## Abbreviations

ICD 8: International Classification of Diseases, 8th edition
ICD 10: International Classification of Diseases, 10th edition
ISCED: International Standard Classification of Education
N: Number of units
RR: Relative risk
SD: Standard deviation
T0, T1, T2: Time 0, time 1, time 2

## Code availability

All code used, and additional resources, is available at https://osf.io/fh2sr.
