# Supplementary material for: Parental physical disease severity and severe documented physical child abuse: a prospective cohort study
Source: Eur J Pediatr. 2023 Oct 27;183(1):357–69. doi: 10.1007/s00431-023-05291-8 (PMC10857964; doi:10.1007/s00431-023-05291-8)
Supplement: Supplementary file 1 — Supplementary file1 (DOCX 8 KB) [file 431_2023_5291_MOESM1_ESM.docx]

**Table S1: Baseline characteristics of data for model 1a and 1b**

| **Characteristics** | **Joint Charlson score**  **< 2**  **N: 1,019,456** | **Joint Charlson score**  **≥ 2**  **N: 140,854** | **Total population**  **N: 1,160,310** |
| --- | --- | --- | --- |
| Child age, y, median (quartile 1 ; quartile 3)^a^ | 10.5 (5.0 ; 14.7) | 8.6 (3.2 ; 13.5) | 10.3 (4.8 ; 14.6) |
|  |  |  |  |
| Calendar year group, n (%) |  |  |  |
| 1997-2002 | 282672 (28%) | 48865 (35%) | 331537 (29%) |
| 2003-2009 | 311315 (31%) | 42512 (30%) | 353827 (31%) |
| 2010-2018 | 425469 (42%) | 49477 (35%) | 474946 (41%) |
|  |  |  |  |
| Mean parental age, y, mean (SD) | 41.46 (6.84) | 40.23 (6.92) | 41.31 (6.86) |
|  |  |  |  |
| Neighborhood resources, thousand Euros, mean (SD) | 120.45 (41.46) | 117.14 (40.9) | 120.05 (41.4) |
|  |  |  |  |
| Number of children in family, n (%) |  |  |  |
| One child | 389372 (38%) | 49846 (35%) | 439218 (38%) |
| Two children | 453895 (45%) | 64622 (46%) | 518517 (45%) |
| Three to five children | 174081 (17%) | 26086 (19%) | 200167 (17%) |
| Six or more children | 2108 (0.2%) | 300 (0.2%) | 2408 (0.2%) |
|  |  |  |  |
| Abuse of parent as a child, n (%) |  |  |  |
| No maltreatment or neglect | 953538 (94%) | 128472 (91%) | 1082010 (93%) |
| Maltreatment or neglect, one or both parents | 65918 (6.5%) | 12382 (8.8%) | 78300 (6.8%) |
|  |  |  |  |
| Immigration background (ethnicity), n, (%) |  |  |  |
| No foreign parents | 895582 (88%) | 121907 (87%) | 1017489 (88%) |
| One or more foreign parents | 123874 (12%) | 18947 (14%) | 142821 (12%) |
|  |  |  |  |
| Status as refugee, n, (%) |  |  |  |
| Not in need of protection | 1014245 (99%) | 139871 (99%) | 1154116 (99%) |
| In need of protection | 5211 (0.5%) | 983 (0.7%) | 6194 (0.5%) |
|  |  |  |  |
| Reconstituted family, n, (%) |  |  |  |
| Living with biological parent(s) | 913278 (90%) | 127919 (91%) | 1041197 (90%) |
| Living with one or more unrelated adults | 78355 (7.7%) | 10028 (7.1%) | 88383 (7.6%) |
| Adopted or in foster care | 27823 (2.7%) | 2907 (2.1%) | 30730 (2.7%) |
|  |  |  |  |
| Family highest education, n, (%) |  |  |  |
| Primary or secondary education | 580985 (57%) | 87128 (62%) | 668113 (58%) |
| Tertiary education or higher | 438471 (43%) | 53726 (38%) | 492197 (42%) |
|  |  |  |  |
| Income, thousand Euros, mean (SD) | 148.25 (260.57) | 131.73 (199) | 146.24 (253.95) |
|  |  |  |  |
| Parental psychiatric disease, n, (%) |  |  |  |
| No psychiatric disease | 970166 (95%) | 129763 (92%) | 1099929 (95%) |
| Any psychiatric disease except substance abuse | 49290 (4.8%) | 11091 (7.9%) | 60381 (5.2%) |
|  |  |  |  |
| Inter-parental violence, n, (%) |  |  |  |
| No interparental violence | 1016052 (99%) | 140245 (99%) | 1156297 (99%) |
| Interparental violence | 3404 (0.3%) | 609 (0.4%) | 4013 (0.4%) |
|  |  |  |  |
| Parental substance abuse, n, (%) |  |  |  |
| No parental substance abuse | 1007276 (99%) | 135112 (96%) | 1142388 (99%) |
| Any parental substance abuse | 12180 (1.2%) | 5742 (4.1%) | 17922 (1.5%) |

^a^Rounded to first decimal
