# Supplementary material for: Parental physical disease severity and severe documented physical child abuse: a prospective cohort study
Source: Eur J Pediatr. 2023 Oct 27;183(1):357–69. doi: 10.1007/s00431-023-05291-8 (PMC10857964; doi:10.1007/s00431-023-05291-8)
Supplement: Supplementary file 2 — Supplementary file2 (DOCX 10 KB) [file 431_2023_5291_MOESM2_ESM.docx]

**Table S2: Baseline characteristics of one of five imputed datasets, no missings because of the imputations**

| **Characteristics** | **Joint Charlson score**  **< 2**  **N: 49,140** | **Joint Charlson score**  **≥ 2**  **N: 860** | **Total population**  **N: 50,000** |
| --- | --- | --- | --- |
| Child age, y, median (quartile 1 ; quartile 3)^a^ | 0.4 (0.2 ; 7.2) | 0.8 (0.3 ; 10.9) | 0.4 (0.2 ; 7.3) |
|  |  |  |  |
| Calendar year group, n (%) |  |  |  |
| 1997-2002 | 28873 (59%) | 515 (60%) | 29388 (59%) |
| 2003-2009 | 9486 (19%) | 129 (15%) | 9615 (19%) |
| 2010-2018 | 10781 (22%) | 216 (25%) | 10997 (22%) |
|  |  |  |  |
| Mean parental age, y, mean (SD) | 34.6 (6.56) | 37.64 (7.25) | 34.65 (6.59) |
|  |  |  |  |
| Neighborhood resources, thousand Euros, mean (SD) | 108.62 (38.43) | 109.86 (41.61) | 108.64 (38.49) |
|  |  |  |  |
| Number of children in family, n (%) |  |  |  |
| One child | 21637 (44%) | 402 (47%) | 22039 (44%) |
| Two children | 19198 (39%) | 315 (37%) | 19513 (39%) |
| Three or more children | 8305 (17%) | 143 (17%) | 8448 (17%) |
|  |  |  |  |
| Parental maltreatment in childhood, n (%) |  |  |  |
| No maltreatment or neglect | 45941 (94%) | 810 (94%) | 46751 (94%) |
| Maltreatment or neglect, one or both parents | 3199 (6.5%) | 50 (5.8%) | 3249 (6.5%) |
|  |  |  |  |
| Immigration background (ethnicity), n, (%) |  |  |  |
| No foreign parents | 38262 (78%) | 682 (79%) | 38944 (78%) |
| One or more foreign parents | 10878 (22%) | 178 (21%) | 11056 (22%) |
|  |  |  |  |
| Status as refugee, n, (%) |  |  |  |
| Not in need of protection | 47677 (97%) | 847 (99%) | 48524 (97%) |
| In need of protection | 1463 (3.0%) | 13 (1.5%) | 1476 (3.0%) |
|  |  |  |  |
| Reconstituted family, n, (%) |  |  |  |
| Living with biological parent(s) | 46337 (94%) | 802 (93%) | 47139 (94%) |
| Living with one or more unrelated adults | 1889 (3.8%) | 39 (4.5%) | 1928 (3.9%) |
| Adopted or in foster care | 914 (1.9%) | 19 (2.2%) | 933 (1.9%) |
|  |  |  |  |
| Family highest education, n, (%) |  |  |  |
| Primary or secondary education | 31092 (63%) | 514 (60%) | 31606 (63%) |
| Tertiary education or higher | 18048 (37%) | 346 (40%) | 18394 (37%) |
|  |  |  |  |
| Family income, thousand Euros, mean (SD) | 111.7 (149.97) | 123.56 (111.74) | 111.9 (149.4) |
|  |  |  |  |
| Parental psychiatric disease, n, (%) |  |  |  |
| No psychiatric disease | 47276 (96%) | 808 (94%) | 48084 (96%) |
| Any psychiatric disease except substance abuse | 1864 (3.8%) | 52 (6.1%) | 1916 (3.8%) |
|  |  |  |  |
| Inter-parental violence, n, (%) |  |  |  |
| No interparental violence | Removed^b^ | Removed^b^ | 49981 (99%) |
| Interparental violence | Removed^b^ | Removed^b^ | 19 (0.0%) |
|  |  |  |  |
| Parental substance abuse, n, (%) |  |  |  |
| No parental substance abuse | 48764 (99%) | 836 (97%) | 49600 (99%) |
| Any parental substance abuse | 376 (0.8%) | 24 (2.8%) | 400 (0.8%) |

^a^Rounded to first decimal

^b^Rules from Statistics Denmark stipulates that cells smaller than 5 are removed to avoid risk of identification.
